# Endoscopic sequential therapy for portal hypertension: Concept and clinical efficacy^☆^

**DOI:** 10.1016/j.livres.2020.12.004

**Published:** 2020-12-31

**Authors:** Xing Wang, Bin Wu

**Affiliations:** a Department of Gastroenterology, The Third Affiliated Hospital of Sun Yat-sen University, Guangzhou, China; b Guangdong Province Engineering and Technology Research Center of Digestive Endoscopy, Guangzhou, China

Variceal bleeding is the most dreadful complication of liver cirrhosis and adversely affected 6.8 million people worldwide in 2017, with up to 80% 5-year mortality.^1^^,^^2^ Endoscopic therapies have played a pivotal role in the management of variceal bleeding for more than three decades, and in recent years, the rebleeding rate and bleeding-related mortality at 1 year have been reduced from 60% to 30% and 50% to 15–20%, respectively.^3^, ^4^, ^5^ Complete eradication of esophageal varices (EV), which can be obtained by repeated sessions of operational endoscopies, was recommended as the therapeutic goal of endoscopic treatment by recent clinical guidelines.^1^^,^^6^ However, endoscopic treatment for gastric varices (GV) is still challenging, and there is no consensus on the criterion for GV eradication or obliteration. To date, a standard endoscopic protocol integrating treatment for both EV and GV has not been established. Since over 80% of patients admitted for liver cirrhosis in tertiary healthcare centers present with high-risk esophago-gastric varices (EGV) and require endoscopic therapy,^7^ a standard but individualized endoscopic solution for treating both EV and GV is highly needed. Therefore, we herein introduce our systematic solution of endoscopic sequential therapy (EST) in terms of concept, performance and clinical efficacy. This solution integrates EV and GV treatment, emphasizes complete variceal eradication, and aims to sustain variceal remission and improve long-term prognosis.

## 1 Concept of EST

The concept of EST is derived from endoscopic combination therapy. Endoscopic band ligation (EBL) is currently the mainstay for the treatment of EV and gastroesophageal varices (GOV) proximal to the esophageal-gastric (E-G) junction, while endoscopic injection sclerotherapy (EIS) is not the priority choice but is still useful for small remnant varices as well as on-site EBL failure in some patients.^8^^,^^9^ Regarding GV, glue injection with N-butyl-2-cyanoacrylate or 2-octyl-cyanoacrylate can be used in most patients except those with sole large isolated gastric varices (IGV).^1^ The recommendations of current guidelines and consensus regarding endoscopic treatment for EV and variant types of GV are summarized in Table 1.^1^^,^^6^^,^^10^, ^11^, ^12^, ^13^ However, most previous studies analyzed data on EV alone, and few studies evaluated patients undergoing endoscopic treatment for both EV and coexisting junctional or fundal GV. Considering that GV occurred in 50% of cirrhotic patients and contributed to 10–20% of variceal bleeding cases, complete occlusion of both EV and GV was supposed to be a more ideal endoscopic endpoint than EV eradication alone.^11^^,^^14^^,^^15^ Therefore, a solution integrating EV and GV therapy was proposed, and the performance principle was based on clinical practice guidelines in combination with our local expertise.^6^^,^^11^^,^^12^^,^^16^, ^17^, ^18^

**Table 1:** Recommendations of practice guidelines and consensus regarding endoscopic options for treating different types of EGV in secondary prophylaxis.

Guidelines/consensus (year)	Recommendations for EV	Recommendations for GV			
		GOV1	GOV2	IGV1	IGV2
AASLD guidance (2017)^1^	EBL	EBL or ECI	ECI^a^	ECI^a^	EBL, ECI or others^b^
EASL guideline (2018)^10^	EBL	EBL or ECI	ECI	ECI	N/A
UK guideline (2015)^11^	EBL	EBL or ECI^c^	ECI	ECI	ECI
Baveno VI consensus (2015)^12^	EBL	EBL or ECI	ECI	ECI	ECI
Japanese guideline (2016)^6^	EIS, or EIS + EBL	ECI	ECI	ECI	N/A
Chinese guideline (2016)^13^	EBL, EIS or EBL + EIS	ECI, EBL or EIS	ECI, EBL or EIS	N/A	N/A
Our EST principle (2020)	EBL, EIS or EBL + EIS	EBL or ECI	ECI	ECI	N/A

Abbreviations: AASLD, American Association for the Study of Liver Diseases; EASL, European Association for the Study of the Liver; EBL, endoscopic band ligation; ECI, endoscopic cyanoacrylate injection; EGV, esophago-gastric varices; EIS, endoscopic injection sclerotherapy; EST, endoscopic sequential therapy; EV, esophageal varices; GOV, gastroesophageal varices; GV, gastric varices; IGV, isolated gastric varices; N/A, no formal recommendations available.

a Recommended when transjugular intrahepatic portosystemic shunt (TIPS) or balloon-occluded retrograde transvenous obliteration (BRTO) are not available.

b Endosonographic coil placement and EIS were also recommended.

c Recommended when coexisting with other types of GV.

## 2 Key points in performance and patient follow-up

### 2.1 General principle of performance

EST is a compound strategy of performing repeated endoscopic sessions until variceal eradication is achieved. The procedure was initiated in the early 2000s and evolved over time.^19^^,^^20^ Herein, we introduce the updated version of the procedure developed in our department. The endoscopic options comprise endoscopic cyanoacrylate injection (ECI), EBL and EIS, or any combination thereof. In general, GV proximal to the E-G junction and less than 10 mm in diameter with high-risk bleeding stigmata can be treated properly with either EBL or ECI according to the discretion and preference of operating physicians. For GV distal to the E-G junction, all high-risk GV regardless of size as well as those ≥10 mm in diameter should be treated with ECI at the initial visit. What follows are repeated EBL sessions for EV or additional ECI sessions for GV every 4 weeks. The follow-up interval may be extended to 6–12 weeks when the ECI session is performed due to a longer time for the reassessment of GV obliteration after glue injection.^21^ When EV became discontinuous or were surrounded by scars where EBL was inapplicable, EIS was performed for the remnant high-risk EV to achieve complete variceal eradication. Non-selective beta-blockers (NSBBs) in combination with endoscopic therapy have been recommended as standard treatment for patients with EV, whether on primary prophylaxis or secondary prophylaxis.^1^ Although the evidence is not as strong as for EV, NSBBs are also suggested for GV patients on primary prophylaxis. However, for secondary prophylaxis, this medication may have beneficial effects only for GOV1 patients and should be initiated in a timely manner after the acute bleeding phase.^1^^,^^3^

### 2.2 ECI procedure

ECI was performed using a mixture of N-butyl-2-cyanoacrylate and lipiodol at a 1:1 ratio. Due to multi-origin afferents to the GV, the selection of the injection site is challenging, especially for GOV2 and IGV type vessels.^22^ In principle, for GOV vessels, the injection site should be distal to the cardia and a short distance away from the rupture or clot point if any. Regarding IGV vessels, for which it is difficult to determine blood flow, the injection should be located in the middle of the bulging part of the varix. Moreover, endoscopic accessibility should also be considered to finally determine the injection site.^23^ The dosage of the mixture was determined by the operating endoscopist depending on the type and size of the GV. Severe complications associated with ECI include systemic embolization and early rebleeding during glue extrusion, which occurred in approximately 3% and 1% of patients, respectively. Injection at a constant speed and restricted mixture volume may help reduce the risk of complications.^21^

### 2.3 EIS procedure

EIS was performed using 1% lauromacrogol as the sclerosant for injection. No more than 5 mL of the agent was injected at each site, and the total amount did not exceed 20 mL for each session. Severe EIS-induced complications include esophageal stricture, esophageal perforation, bronchoesophageal fistula, and mediastinum-related complications.^8^^,^^21^ However, we did not observe the above complications in patients receiving EST in our endoscopy center, which may be because we performed only intravascular sclerosant injection, and a small amount of sclerosant was injected.

### 2.4 EBL procedure

EBL was performed using multiband ligators. The procedure started at or just below the E-G junction, and each varix was ligated in a step-ladder pattern, but no more than 7 bands were used for each session. In contrast to EIS, complications of EBL were usually mild and did not require specific medical treatment. However, post-band ulcer bleeding was the major adverse event, and it occured in 2.3–7.3% of patients.^24^ Intravenous proton pump inhibitors after the EBL procedure and sufficient doses of NSBBs may help prevent ulcer bleeding.^21^^,^^24^

### 2.5 Post-therapeutic follow-up

**Fig. 1:**
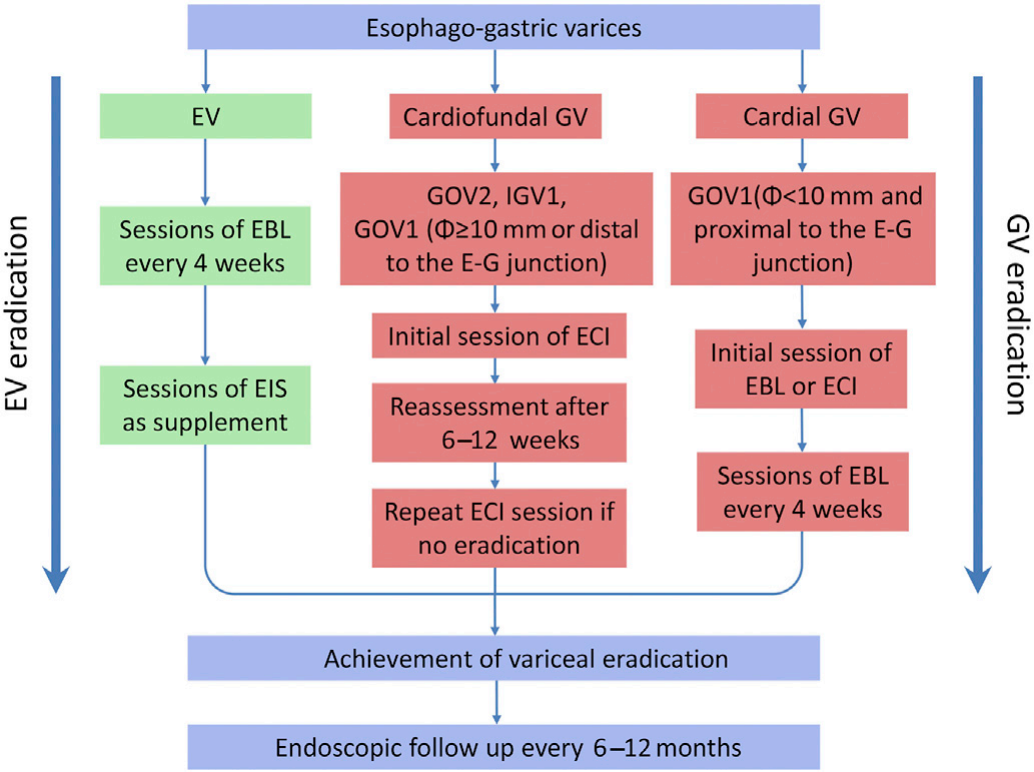
**The updated workflow of EST and subsequent follow-up.** Abbreviations: EBL, endoscopic band ligation; ECI, endoscopic cyanoacrylate injection; E-G junction, esophageal-gastric junction; EIS, endoscopic injection sclerotherapy; EST, endoscopic sequential therapy; EV, esophageal varices; GOV, gastroesophageal varices; GV, gastric varices; IGV, isolated gastric varices.

After the achievement of EGV eradication, we followed up with the patients at 6 months for the first visit and then at every 6–12 months for subsequent visits. When high-risk varices recurred, additional endoscopic treatment was used, and the specific option was selected at the discretion of physicians. Furthermore, the use of NSBBs should be maintained in a long term after EGV eradication to prevent rebleeding and variceal recurrence.^25^ The schematic workflow of EST is shown in Fig. 1.

## 3 Effect of EST on the variceal eradication rate

### 3.1 EV eradication

Endoscopic therapy is effective in eradicating EV. The reported eradication rate of EV varies between 40% and 100% for EBL alone, EIS alone, or their combination.^20^^,^^26^^,^^27^ The rate may be higher when both endoscopic therapy and NSBBs are utilized. From the data of an observational cohort in which EST was applied in secondary prophylaxis patients, we found that the eradication rate was 85.4%, which is higher than most of prior evidence. EBL is considered to be equivalent to EIS in terms of the eradication rate, but the recurrence rate of post-therapeutic varices is much higher.^6^ Fortunately, additional EBL sessions can treat the recurrent varices in this situation with satisfactorily low procedure-related complications.^3^

### 3.2 GV eradication

Regarding endoscopic treatment of GV, in most patients with GOV1, GOV2 and overlapping GOV and IGV1, endoscopy therapy can be used, but the procedures are highly variant among institutions.^15^^,^^18^^,^^28^ GOV are considered an extension of EV and are suggested to be managed similar to EV, but EBL on large GOV may result in fatal recurrent GV bleeding.^1^ According to our experiences, only small-medium GOV (formation of varices F1–F2) located proximal to the E-G junction are eligible for EBL, whereas large GV (formation of varices F3), regardless of location and bleeding stigmata, should be treated with obturation therapy with tissue glue.^29^ Moreover, the criterion for the treatment endpoint has not been clearly defined. It is important to note that, for most patients, complete eradication with flattened or minimal GV upon air inflation may not be practical. Instead, a complete venous obturation, presented as the consolidation of the varices after ECI, should be achieved and is sufficient to prevent further rebleeding.^30^ It is worth noting that glue injection on GV is demanding, while incomplete obturation is associated with severe rebleeding events either from residual varices or during glue excretion.^29^ Endoscopic ultrasound (EUS) can be used to examine the whole picture of collateral vessels around gastric fundus so that EUS-assisted glue injection can be more precise. Recent evidence has indicated the effectiveness of EUS in the selection of injection points during the procedure as well as the evaluation of the effect of intravascular obturation.^31^

## 4 Clinical effect of EST on rebleeding, mortality and variceal recurrence

### 4.1 Effect of EST on variceal rebleeding and mortality

As many as 60% of patients will experience rebleeding after the first episode of variceal bleeding without appropriate secondary prophylaxis. Endoscopic advances during past decades have substantially reduced the rebleeding rate to 30% at 1 year, while the 1-year mortality is approximately 30% for patients with acute bleeding and approximately 20% for patients on secondary prophylaxis.^4^^,^^5^^,^^32^^,^^33^ Limited data have shown that endoscopic variceal eradication can reduce the rebleeding rate to less than 10% at 1 year.^16^^,^^20^ Among patients undergoing EST for secondary prophylaxis, our recent study demonstrated that the 1-year rebleeding rate was 14.9% and 4.0% before and after variceal eradication, respectively, whereas the 1-year and 5-year mortality rates were 0.9% and 9.3%, respectively (unpublished data). Variceal eradication through EST can further reduce rebleeding based on routine endoscopic therapy and is associated with improved long-term prognosis, although the findings need external validation in further multicenter studies with large sample sizes.

### 4.2 Effect of EST on variceal recurrence

EBL is superior to EIS in terms of obtaining acute hemostasis and preventing rebleeding.^1^^,^^3^^,^^11^ However, EBL is also associated with a higher recurrence rate because banding does not affect the feeding vessels of varices, including periesophageal and paraesophageal varices.^9^^,^^20^^,^^34^ Recent data suggested that combining EBL and EIS might decrease the risk of variceal recurrence and achieve variceal eradication faster than EBL or EIS alone.^3^^,^^9^ Although EBL-EIS combination therapy is not recommended by practice guidelines as standard treatment for primary or secondary prophylaxis,^11^^,^^12^ a recent randomized controlled trial indicated that there was no significant difference between the combination therapy and EBL alone regarding complications, variceal recurrence and long-term prognosis, but the combination therapy spared endoscopic time and sessions at similar cost.^34^ It is interesting to note that in this aforementioned study, a very small amount of sclerosant (no more than 2 mL of 5% ethanolamine oleate) was injected into each varix, which is similar to our EST practice. In contrast, data issuing gastric variceal recurrence after ECI are limited, and a recent retrospective study from the United States reported rates of 27% and 46% at 1 year and 2 years, respectively.^30^ EUS is reported to be useful to obliterate deeper and invisible varices and assess variceal recurrence after endoscopic eradication, but additional resources and expertise are required. Debate remains on whether direct-vision endoscopy or sonographic endoscopy is the best choice for eradicating varices, and more randomized controlled studies are expected.^31^

## 5 Clinical effect of EST on portal pressure and hemodynamics

Previous studies have demonstrated that both EBL and EIS may cause an increase in the hepatic venous pressure gradient (HVPG).^34^^,^^35^ In one randomized study, HVPG in the EBL group returned to baseline levels within 48 h after the treatment, but in the EIS group, the rise in HVPG sustained during the 5-day study period.^35^ The local effect of EBL on the mucosa and submucosa is presumed to be the reason for the transient increase in portal pressure, and the HVPG increase in the EIS group may be attributed to the systemic effect of occlusion and the endovascular effect.^34^ Considering that EIS is only occasionally used for remnant varices and the volume of sclerosant injected is small for the EST procedure, there should be no long-term effect on HVPG in patients receiving either EBL or EBL plus EIS treatment. On the other hand, although ECI has greatly decreased the rebleeding rate in secondary prophylaxis, no studies have directly investigated portal pressure or HVPG changes after treatment. In theory, intravascular injection with venous occlusion will result in elevated portal pressure; however, the glue would clot into hard matter and be excreted gradually within weeks to months after the treatment.^27^ In contrast to balloon-occluded retrograde transvenous obliteration (BRTO) with persistent elevated portal pressure, ECI is unlikely to increase portal pressure in a long-term setting.^36^ Apart from portal pressure, Doppler ultrasound investigations did not find significant changes in the direction and velocity of venous blood flow after EBL or EBL-EIS combination therapy.^37^^,^^38^ In addition, sonographic studies measuring cardiac output and mean arterial pressure did not find any significant change after initial or repeated EBL sessions. Therefore, EBL is presumed to have a trivial effect on systemic circulation. When measuring blood flow of the portal vein and superior mesenteric artery, an approximately 20% increase was noted, but the flow returned to the baseline level after repeated sessions. Hence, it may be concluded that EBL has a slightly transient effect on splanchnic hemodynamics.^39^ Recently, a novel technique for EUS-guided portal pressure measurement has provided a more convenient and direct way to evaluate portal venous pressure, while strong correlations were noted between EUS-guided and traditional percutaneous pressure measurements in an animal model study.^40^^,^^41^ More data from large-scale multicenter studies are highly expected before the potential widespread use of this technique in clinical practice.

In summary, EST is a rational and effective modality to eradicate EGV and improve patient prognosis. Meanwhile, the clinical impact of postoperative variceal recurrence in long-term follow-up awaits unveiling in the future. Portal and splanchnic hemodynamic alterations after sole glue injection or combination endoscopic therapy are unclear, and further investigations are greatly needed.

## Authors’ contributions

X. Wang wrote the manuscript. B. Wu provided critical revision of the manuscript and supervised the work.

## Declaration of competing interest

The authors declare that they have no conflict of interest.
